# Social Media Strategies for Health Promotion by Nonprofit Organizations: Multiple Case Study Design

**DOI:** 10.2196/15586

**Published:** 2020-04-06

**Authors:** Isabelle Vedel, Jui Ramaprasad, Liette Lapointe

**Affiliations:** 1 Department of Family Medicine McGill University Montreal, QC Canada; 2 Lady Davis Institute, Jewish General Hospital Montreal, QC Canada; 3 Desautels Faculty of Management McGill University Montreal, QC Canada

**Keywords:** neoplasm, social media, information technology, organizations, nonprofit, cancer

## Abstract

**Background:**

Nonprofit organizations have always played an important role in health promotion. Social media is widely used in health promotion efforts. However, there is a lack of evidence on how decisions regarding the use of social media are undertaken by nonprofit organizations that want to increase their impact in terms of health promotion.

**Objective:**

The aim of this study was to understand why and how nonprofit health care organizations put forth social media strategies to achieve health promotion goals.

**Methods:**

A multiple case study design, using in-depth interviews and a content analysis of each social media strategy, was employed to analyze the use of social media tools by six North American nonprofit organizations dedicated to cancer prevention and management.

**Results:**

The resulting process model demonstrates how social media strategies are enacted by nonprofit organizations to achieve health promotion goals. They put forth three types of social media strategies relative to their use of existing information and communication technologies (ICT)—replicate, transform, or innovate—each affecting the content, format, and delivery of the message differently. Organizations make sense of the social media innovation in complementarity with existing ICT.

**Conclusions:**

For nonprofit organizations, implementing a social media strategy can help achieve health promotion goals. The process of social media strategy implementation could benefit from understanding the rationale, the opportunities, the challenges, and the potentially complementary role of existing ICT strategies.

## Introduction

### Background

Nonprofit organizations have always played an important role in health promotion, such as advertising campaigns using billboards [1], radio [2], or television [3]. However, many health promotion programs run by nonprofit organizations have difficulty achieving success. This can be attributed to challenges with disseminating information to the appropriate target group, often because the target audience is not easily identifiable [4], or individuals ignoring information and not feeling engaged [1].

As a complement to more traditional information and communication technologies (ICT), social media is creating opportunities to address these challenges. Social media “encompasses a wide range of online, word-of-mouth forums” [5] and is characterized by its interactive and digital nature [6]. Nonprofit organizations are increasingly relying on social media to effectively design health promotion strategies [7-9] and to facilitate the reach of word of mouth [10], although some such organizations are not necessarily leveraging all the power social media can offer [11].

To date, research has mainly examined patients’ and professionals’ motives, barriers, and facilitators to the use of social media [12-15], as well as its impacts, both positive and negative [16]. On the one hand, social media has positive impacts for patients, such as enabling them to share experiences, seek information and opinions, engage with peers and providers, and belong to a community [14,16-19]. This, in turn, can improve patients’ sense of participation, motivation, autonomy, empowerment, perceived self-efficacy engagement in decision making, emotional support, and self-care [14,16-18]. These factors associated with social media can contribute to a positive impact on patient health: if social media enables patients to be more engaged in their health, they will change their behavior more easily [17]. However, there is also the risk of unreliable and incorrect health information provided by the community for the community [20].

### Objectives

What is not clear from this literature is how decisions regarding the use of social media are undertaken by nonprofit organizations that want to increase their impact in terms of health promotion. Our study, conducted in the context of cancer, aims at understanding why and how nonprofit organizations develop social media strategies, with the goal of eliciting how such organizations can successfully leverage social media. Looking at the use of social media from the organizational perspective allows us to understand the characteristics of the social media strategies that are utilized by nonprofit organizations and to identify how social media may help organizations attain their goals of health promotion. This understanding is critical in providing guidance on how such organizations can leverage social media and manipulate the factors or change the conditions of their social media use to ultimately increase their impact on health promotion.

## Methods

### Design

We conducted a multiple case study to examine how six North American nonprofit cancer organizations engage in the use of social media for health promotion.

### Theoretical Framework

Our study is based on the organizing vision theoretical lens [21], which leverages the concept of mindfulness. In a learning organization, there is a commitment on learning and communication. The leadership of such organizations associate learning to organizational success and to sustaining a supportive learning culture [22]. Organizational mindfulness is “a combination of ongoing scrutiny of existing expectations, continuous refinement and differentiation of expectations based on newer experiences, willingness and capability to invent new expectations that make sense of unprecedented events” [23]. Hence, although a learning organization is focused on ensuring *organizational memory*, the construct of mindfulness embeds, in addition, a prospective and innovative perspective. The concept of mindfulness has proven to be useful to shed light not only on the organizational adoption of ICT innovations but also to inform how organizations can chart a successful course for ICT implementations, by remaining vigilant vis-à-vis ICT evolution [21,24-27]. To the best of our knowledge, this lens has not been used to examine social media.

Mindful behaviors of organizations mean openness to new information and awareness of multiple perspectives [28]. Mindful organizations are described as those that make appropriate interpretations of their nature and needs and respond adaptively to changes in their environment [29]. Rooted in this perspective, the organizing vision is a lens that helps explain how organizations can implement ICT innovations mindfully [30]. It shows how mindful organizations can become increasingly attentive to their idiosyncrasies and environment, to make the most of their ICT investments [31]. Mindfully innovating with ICT means that the organization “attends to an IT [Information Technology] innovation with reasoning grounded in its own organizational facts and specifics” [30], whereas innovating mindlessly with ICT refers to the instance where “a firm’s actions betray an absence of such attention and grounding” [30].

Leveraging on the organizing vision lens, we adopted a theory-building approach, based on a multiple case study design [32,33].

### Cases

The six cases in this study were selected based on a maximum variation sampling strategy [34] and focused on organizations using social media for cancer prevention and management (Table 1), a major public health issue in our society [35]. A detailed description of the key characteristics of each case is provided in Multimedia Appendix 1, including the rationale of social media use and the ICT and social media tools used.

**Table 1:** Case characteristics and social media tools used.

Cases	Characteristics	Social media tools
Case 1: Breast Cancer Action	Country: United States, disease type: breast cancer, year founded: 1990, and number of employees: 8	Facebook, Twitter, YouTube, and blog
Case 2: Breast Cancer Society	Country: Canada, disease type: breast cancer, year founded: 1991, and number of employees: 5	Facebook, Twitter, LinkedIn, and YouTube
Case 3: Breast Cancer Foundation	Country: Canada, disease type: breast cancer, year founded: 1986, and number of employees: 197	Facebook, Twitter, LinkedIn, YouTube, Flickr, and blog
Case 4: Us Too International	Country: United States, disease type: prostate cancer, year founded: 1990, and number of employees: 5	Facebook, Twitter, LinkedIn, YouTube, Wikis, Groupon, and blog
Case 5: Prostate Cancer Foundation	Country: United States, disease type: prostate cancer, year founded: 1993, and number of employees: 30	Facebook, Twitter, LinkedIn, YouTube, and blog
Case 6: Pints for Prostate	Country: United States, disease type: prostate cancer, year founded: 2008, and number of employees: 2	Facebook, Twitter, Flickr, and Vimeo

### Data Sources and Data Collection

We triangulated our data sources: semistructured interviews with key informants, analysis of the documentation (eg, documentation describing the organization, reports, and newsletters), and qualitative content analysis of the websites and the social media tools used (eg, Facebook, Twitter, and YouTube). In each organization, we conducted semistructured interviews with the chief executive officer or the person responsible for the social media development and use (ie, the key informants) in winter 2008-2009 [34]. These respondents had a thorough knowledge of the origins, implementation, use, barriers, and enabling factors of ICT and social media usage in their respective organizations. Our interview guide (Multimedia Appendix 2) was validated and refined using four pilot interviews. The interviews lasted 1 hour on average and were recorded and transcribed verbatim in their entirety. In addition, we asked our participants to provide relevant documentation. We also collected data from the social media tools across 1 calendar year (2012), to minimize biases. In the end, for each organization, we created a data dossier that provides a structured summary of the characteristics of the organization, content of the website, and social media tools (Multimedia Appendix 2). The overall data collection process resulted in several hundred pages of transcripts and social media content data dossiers.

### Analysis

Analytic induction was deemed to provide the best analytic strategy for this study [34,36-38]. Indeed, analytic induction begins with a deductive phase [34,39], which allows for the use of existing theory, and is followed by an inductive phase that allows for new insights to emerge from the data. Following the data collection process, we proceeded with the first round of coding of the social media data dossier and interview transcripts. Our initial codes were deductively based on the categories derived from our organizing vision theoretical lens to understand how organizations learned to best exploit social media through comprehension, adoption, implementation, and assimilation. Next, we proceeded to a round of open coding and identified new themes (eg, actions, tools, and practices put in place). Afterward, following axial coding, codes with the same content and meaning were grouped in higher-level categories (eg, rationale for using social media tools, complementarity with existing ICT, and challenges). Finally, through selective coding, we linked the resulting categories to the main category (eg, strategies). The analysis of the documentation was used to provide additional information and to corroborate and validate the information gathered via the interviews and the social media data dossier. During the overall process of data coding, as a team, we reviewed and discussed the codification of data until we had reached a consensus; this helped eliminate any potential discrepancy. Examples of codes are provided in Multimedia Appendix 3. N’Vivo 9 (QRS International Pty Ltd) was used to support the coding and analysis of the transcripts.

The analysis followed an iterative process, from reading the data to the data analysis multiple times. This iteration allowed a progressive theory development process with an increasing level of abstraction [40], that is, the creation of a shared understanding that forms a coherent structure, a unified whole. This was repeated until theoretical saturation (ie, the point at which additional analysis repeatedly confirmed the interpretations already made) [41]. Following this iterative analysis process, we developed our process model of social media strategies for health promotion by nonprofit organizations.

## Results

### Overall Findings

Overall, the analysis allowed us to build upon the four pillars of our organizing vision theoretical lens. First, we saw how organizations need to *comprehend* how social media can—or cannot—apply to their needs and reality in terms of health promotion. Second, mindful ICT *adoption* signifies the ability “to anchor the decision in local particulars, rather than simply follow the lead and public rationales or prior adopters” [31]. Third, in *implementing* social media, organizations have to be sensitive to their reality and idiosyncrasies. Finally, the mindfulness challenge in *assimilation* is to decide how to optimally integrate social media into everyday operations to have a better impact on health promotion. We provide illustrative quotes in Multimedia Appendix 4 and examples from the data dossier in Multimedia Appendix 5.

The cross-case analysis—of the ICT and social media tools, interviews, and documents—revealed no major variation in the results among cases based on the cancer type they were concerned with, the country the organization is based in, the nature of the social media tools the organization employed, or the organization size. Although some of the larger organizations were able to assign some nonspecialized personnel to their social media activities, these activities mainly consisted of feeding the social media platforms, not developing the social media strategy. The analysis of the data dossiers did not reveal any major differences in why and how nonprofit organizations develop social media strategies.

### Comprehension

Organizations tend to have one or several of the five following *rationales* for the adoption of social media in health promotion:

1. Creating awareness: Organizations use social media tools to advertise about the disease and to promote healthy behaviors (eg, screening). Social media can be particularly useful to provide information that can be tailored to a specific audience and to reach people who are not voluntarily seeking the information (see quotes 1-3 in Multimedia Appendix 4).
2. Educating: Social media tools can provide up-to-date information on the disease (eg, risk factors) and can enable end users (patients, families, and significant others) to make better informed decisions (eg, about treatment options—see quotes 4 and 5).
3. Providing a forum to interact and support: Social media tools such as blogs, forums, or tweets allow users to get advice from the organization and to facilitate user interactions among themselves for support (see quotes 6-8).
4. Advocating: Social media tools are also, at times, used to play an activist role in relation to the organizations’ missions (see quotes 9 and 10).
5. Raising funds: Social media could be a way to facilitate communications and connections with donors (see quotes 11-13). Organizations may also track and report on social media metrics (eg, number of tweets and retweets), for the purposes of board and donor accountability.

In addition, six important *opportunities* associated with the use of the social media tools were identified:

1. Ease-of-use: Social media tools are perceived to be easy to use and provide the opportunity to easily reach a large number of individuals, as evidenced by the number of fans, followers, posts, and blogs (see quote 14 and Multimedia Appendix 5).
2. Low cost: Social media is seen as a low-cost tool compared with traditional marketing tools. For small organizations with limited budgets, such low-cost tools provide new opportunities to communicate and provide information (see quotes 15 and 16).
3. Interactivity with end users: Social media provides a forum for individuals to connect with each other and to engage in more personalized discussions in a timely manner (see quotes 17 and 18). Data show active participation of users (Multimedia Appendix 5) and better effectiveness. For example, end users can follow links and choose the path of information that they would like to explore deeper (see quotes 19 and 20).
4. Flexibility: Social media tools do not impose a strict structure on how the tools are used, how individuals choose to interact and access information using these tools, and how they are integrated with other media (see quotes 21 and 22). This was further evidenced by the links for YouTube videos that were found on many Facebook pages (Multimedia Appendix 5).
5. Status: The use of social media tools was associated with a desire for status differentiation and perceptions of popularity, trendiness, reputation, efficiency, etc (see quotes 23 and 24).
6. Virability: Social media’s increased ease in spreading information compared with more traditional ICT—what we call virability—was evidenced by the ability to repost information on Facebook and Twitter (Multimedia Appendix 5), sometimes through mobile devices (see quotes 25 and 26).

### Adoption

To maximize the impact, all six organizations used social media tools in addition to some ICT tools (eg, webpages and electronic newsletters) and even more traditional communication tools (eg, posters, magazine, and television advertisements; see quotes 27, 28, and 29 and Multimedia Appendix 5). They saw social media as a way to add to what they were already doing, to give more strength to their activities, and to augment and expand the capabilities of the ICT tools (see quotes 30-32). Concretely, analysis revealed three specific social media *strategies*:

1. Replicate: Organizations essentially imitate their existing use of ICTs, but through a different channel to reach a different and broader audience (see quotes 33 and 34).
2. Transform: Organizations use social media for the same purpose as it uses ICT tools, but the message is transformed in the way it is formatted and delivered, to better engage end users (see quotes 35 and 36).
3. Innovate: To truly tap in the soul of social media, organizations modify the message or action for a new purpose, seeking different results. Such a strategy entails, for example, reposting a message, taking advantage of the virability of the media, and using blogs for press conferences or virtual billboards for advertising. Altogether such a strategy may ultimately enable the development of a community (see quotes 37 and 38).

### Implementation

To better take into account the reality of their usage and context, organizations have had to deal with several *challenges*:

- Lack of control: Managing the openness in communication that is enabled through social media (Multimedia Appendix 5) and appropriately monitor the quality, quantity, and format of conversations individuals were having (see quotes 39 and 40). This difficulty concerns both the user contribution and the information that the organization and partners themselves provided (see quotes 41 and 42).
- Technology-related issues: Although user friendly, technology usage introduces challenges such as forced upon updates and characteristics that create limitations (see quotes 43 and 44).
- Diversity of audience: Reaching a wider audience creates challenges in tailoring the message to different communities (eg, an older population and less educated individuals; see quotes 45 and 46 and Multimedia Appendix 5).
- Availability of resources: Finding the resources to develop and manage social media was considered challenging, given the need to find individuals with the expertise in both the content (cancer) and the social media tool. Moreover, there is a need to maintain a social media presence at a high level of interactivity, which requires an extensive amount of time (see quotes 47-50).
- Difficulty in measuring impacts: It is difficult to define relevant indicators of success and objectively assess whether social media use truly helps meet goals (see quotes 51 and 52).

### Assimilation

In assimilation, organizations decide how to optimally integrate the new social media tools into everyday operations.

1. Mindless/mindful: At the onset, organizations did not necessarily adopt or use social media in a well thought-out manner, with clear objectives in mind. Actually, the initial use of social media in most of the organizations was primarily mindless. This was particularly noticeable in the case of two organizations where the decision to use social media was not a planned event and where social media strategies were enacted to seize emergent opportunities (see quotes 53 and 54). The level of mindfulness of social media use by the organizations we studied evolved. With time, some organizations were beginning to reflect more about social media (see quotes 55 and 56). Interestingly, in the organization that was most mindful at the onset, social media usage continued to evolve in the same manner, maintaining a mindful stance (see quote 57).
2. Reactive/proactive: Above and beyond the mindful/mindless stance of the process, our results show that the social media strategies were at times enacted in a reactive manner and at other times in a proactive manner. Social media strategies were initially implemented mainly in a reactive manner (ie, in response to users’ explicit needs; see quote 58). Only one organization exhibited goal-directed behavior and demonstrated anticipation—a proactive orientation—that is, enabling change before such needs are overtly expressed (see quote 59).

### Connecting the Dots

**Figure 1:**
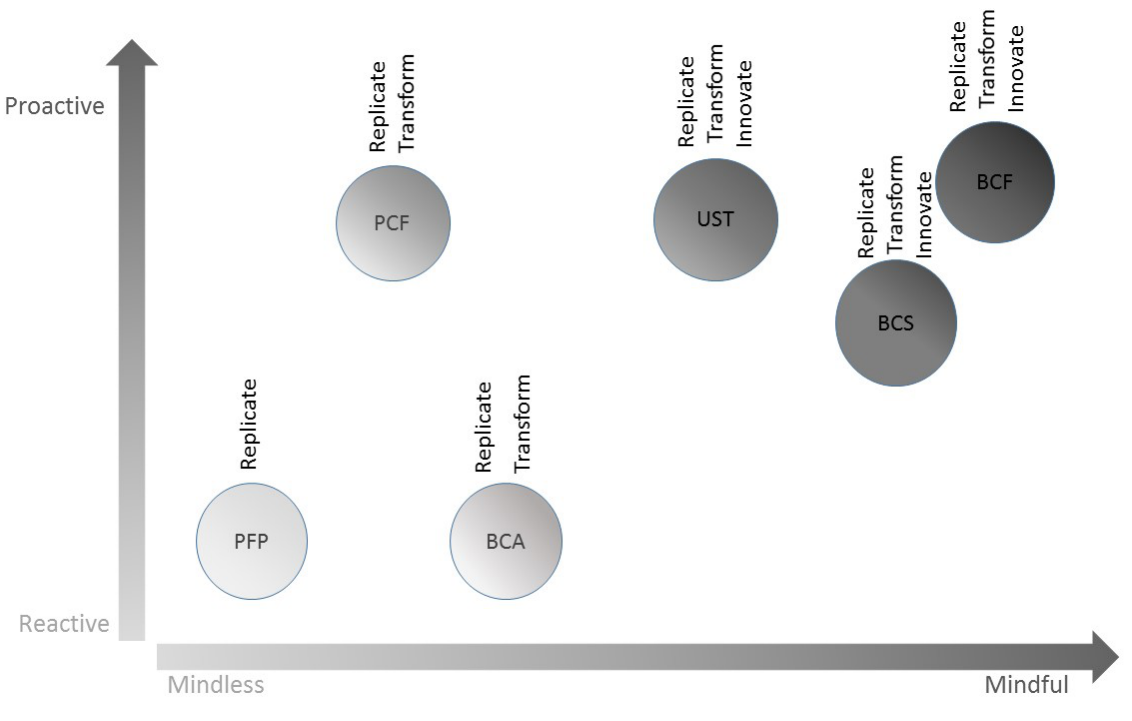
Mindfulness and proactive orientation of the six cases. BCA: Breast Cancer Action; BCF: Breast Cancer Foundation; PCF: Prostate Cancer Foundation; PFP: Pints for Prostate; UsT: Us Too International.

In summary, our data revealed that in addition to considering the level of mindfulness, it was important to consider the proactiveness, or lack thereof, exhibited by the organizations. We linked the strategies put forth by organizations to their overall level of mindfulness and proactive orientation (Figure 1).

We identified three clusters:

1. Cluster 1: The organization exhibits a low level of mindfulness and little proactiveness. The only strategy that was mobilized is this case was replicate. Hence, this organization mostly used social media to carry on the same activities but using social media (see quotes 60-62).
2. Cluster 2: One organization exhibited a fairly low level of mindfulness but a high proactive orientation; another organization exhibited a low proactive orientation but a higher level of mindfulness. In both cases, these organizations leverage social media to transform their message, using the particularities of social media to better engage users (see quotes 63 and 64). Despite the fact that these organizations are not both proactive and mindful, they do appear to derive higher value from their social media strategies in terms of health promotion (see quotes 65 and 66) than organizations exhibiting a low level of mindfulness and little proactiveness (ie, cluster 1).
3. Cluster 3: Organizations exhibit a higher level of mindfulness compared with the other clusters. In all, three organizations did not use social media simply to replicate or to transform their message but most importantly to innovate by leveraging the potential offered by social media (see quote 67 and 68). Not surprisingly, these organizations appear to derive the most value from their involvement in social media (see quotes 69 and 70).

### The Process Model of Social Media Strategies for Health Promotion by Nonprofit Organizations

**Figure 2:**
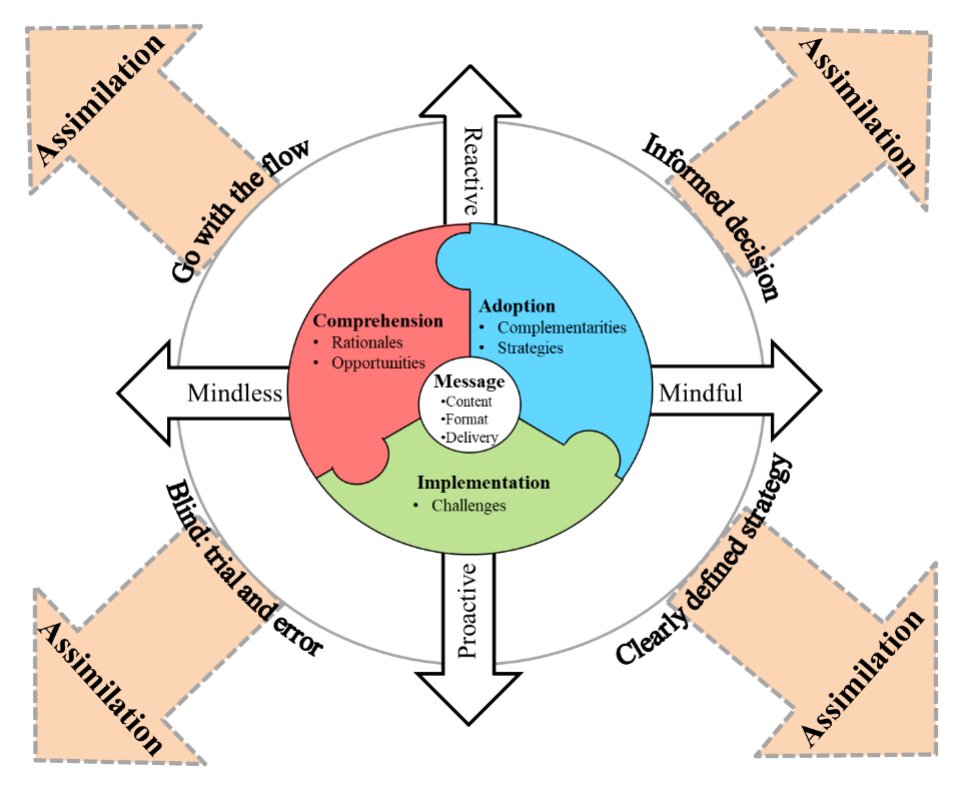
Process model of social media strategies for health promotion by nonprofit organizations.

On the basis of our data analysis and the organizing vision theoretical lens, we developed a process model that reveals the elements and patterns of relationships that underlie the enactment of social media strategies by organizations for health promotion (Figure 2).

It first shows that the four pillars of social media strategy enactment—*comprehension*, *adoption*, *implementation*, and *assimilation*—are not necessarily observed sequentially. Instead, they are intertwined, can occur in any order, and are often iterative. As such, assimilation can occur anywhere in the social media enactment process.

Our model also shows that the organizations need to comprehend the *rationales* and *opportunities* linked with social media tools. They develop their social media *strategies* (replicate, transform, innovate) based on the *complementarities* they seek between existing ICT and social media, which will affect the content, the format, and the delivery of the message (Table 2). Our model also shows that to leverage their social media *strategies*, organizations also need to balance opportunities with the inherent *challenges* of social media.

This social media enactment process is also embedded in the orientation—*proactive* vs *reactive*—and the level of *mindfulness* vs *mindlessness* in which social media strategies are put in place, as illustrated in Table 3.

**Table 2:** Social media strategies: key message characteristics in the synergistic use of information and communication technologies and social media tools.

Strategies	Replicate	Transform	Innovate
Content	Same	Same	Different
Format	Same	Different	Different
Delivery	Different	Different	Different

**Table 3:** Reactive/proactive and mindless/mindful social media strategies enactment.

Orientation	Reactive	Proactive
Mindless	Type 1—go with the flow	Type 2—blind: trial and error
Mindful	Type 3—informed decision	Type 4—clearly defined strategy

When organizations are mindless and reactive (type 1), they generally *go with the flow*, that is, they observe and follow what is happening in the field. When organizations are more proactive, although still mindless (type 2), they do not have a clear plan for their social media strategy. Regardless, they attempt to stay in the forefront of their social media use and iteratively adjust their subsequent social media decisions on a *trial-and-error* basis. When organizations are mindful and reactive (type 3), they are observing others’ usage of social media and assessing its potential value. They then decide whether and how to engage in implementing their social media strategy, thus making an *informed decision* but without a clear and definite plan of action. The final category (type 4) is when organizations are self-aware, staying on the edge, and create a *clearly defined strategy*. They then act with foresight, in a strategic and rational manner, which occurs when organizations are proactive and mindful.

## Discussion

### Principal Findings

Understanding how social media strategies are enacted and how social media can be strategically leveraged at the organizational level is an understudied area of research in health care. Recent work has established the importance of social media for patients and professionals to enable interactions and to access information [14]. We complement this work by looking at social media adoption by nonprofit cancer organizations—institutions that are central in health promotion. The goals of this study were to understand why and how six organizations put forth and enact social media strategies to achieve health promotion goals. Our analysis revealed five main rationales for adoption of social media, as described above, and a process of organizational adoption that we visualize in Figure 2. A key aspect of the all the rationales identified is that they have the common goal of enabling interaction with patients, families, and members of the community for reasons ranging from creating awareness and educating individuals to raising funds for the organizations.

This study adds to the existing literature around patient and professional use of social media [14,42] and extends it by delving deep into the process of adoption of social media by nonprofit organizations. In doing this, we not only look at social media by itself but also its use alongside other ICT tools [43]. To the best of our knowledge, no prior work has taken this approach, which provides an overarching view of the social media adoption process by organizations, a comprehensive understanding of opportunities and challenges associated with adoption of social media, and practical implications for managers who seek to use social media.

One of our key findings in this study is that to leverage their social media strategies, organizations need to balance *opportunities* with the inherent *challenges* of social media, such as lack of control [44], risk of misinformation, lack of privacy, limited audience, usability of social media programs, and the manipulation of identity [17]. With the recent attention to the spread of misinformation on the Web, organizations must understand and implement mechanisms to combat the risks associated with misinformation and privacy. It is critical that information is disseminated from credible sources, such as the organizations that we studied, using tools and technologies that end users, such as patients and their families, can access.

Furthermore, when studying organizational social media use, the question of *how* organizations should communicate with stakeholders is vital [45]. Results from our study suggest that it is imperative to consider the existing ICT when adopting a social media strategy. Our results shows that depending on the *complementarity* sought by the concomitant use of ICT and social media [46], organizations will seek to create the optimal *synergy* between the two strategies when interacting with users, which is consistent with current research findings that suggest that ICT provides most value when combined with other existing resources in the organization [46]. In developing social media *strategies* that take this complementarity into account, organizations must consider the capabilities of the tools along three dimensions: the content, the format, and the delivery [47,48]. Indeed, “...strategies do not need to be drastically overhauled to incorporate social media but merely retooled in framing messages and targeting audiences using the new media” [49].

Overall, although some organizations embrace social media to be at the forefront of innovation to provide health promotion, for others, social media adoption appears to be more of a bandwagon effect. Organizations feel pressure to use social media as they see their competitors and peers using it. In making decisions about social media, organizations face a highly ambiguous environment because of its novelty. Indeed, at the organizational level, the impacts of social media strategies, and their benefits and risks, are still uncertain. Previous research indicates that under high-ambiguity conditions, bandwagon pressures tend to increase [28]. In addition, it has been said that the idea of “mindlessness in innovating with IT [Information Technologies] can reasonably be entertained whenever and wherever its likely rewards outweigh its risks” [30]. However, with time, as the understanding of social media and its role at the organizational level becomes clearer, it is to be expected that organizations would move toward enacting more mindful and proactive social media strategies. Indeed, “mindfulness is not something that an organization possesses: Instead, it is something that emerges in a process of becoming” [50]. Our results suggest that a proactive/mindful stance contributes to improve health promotion.

These results also pave the way for future research, such as testing the model using a larger sample to understand how this process may change depending on the type of organizations (eg, public health agencies, hospitals, private health care organizations, and bigger organization with dedicated staff for the social media activities). Moreover, it would be interesting to take into account the material properties of the social media tools themselves [51-53]. In that perspective, a study of the affordances of each social media tool could be insightful.

### Conclusions

Our process model of social media strategies for health promotion by nonprofit organizations provides a means for managers of nonprofit organizations to understand the rationale of social media strategies and the role that social media can play in health promotion. Our process model can also be used as a guiding framework for nonprofit organizations engaging in social media use for health promotion. These organizations often face the challenge of effectively disseminating information to and engaging with the correct target group, all at low cost. This study provides these organizations with a mechanism for assessing how they can best exploit social media, taking into consideration the opportunities and challenges they face and the complementarities with their existing ICT. Using and understanding these mechanisms can help them create a well-defined strategy that will permit synergies between the existing ICT and social media, so that the use of both sets of tools together will bring in benefits that will surpass the simple sum of each.

## Appendix

Multimedia Appendix 1 Detailed presentation of each case.

Multimedia Appendix 2 Data collection tools.

Multimedia Appendix 3 Coding scheme.

Multimedia Appendix 4 Quotes from key informants.

Multimedia Appendix 5 Data dossier.

## Abbreviations

ICT: information and communication technologies
