# Supplementary material for: Social Media Strategies for Health Promotion by Nonprofit Organizations: Multiple Case Study Design
Source: J Med Internet Res. 2020 Apr 6;22(4):e15586. doi: 10.2196/15586 (PMC7171585; doi:10.2196/15586)
Supplement: Multimedia Appendix 1 [file jmir_v22i4e15586_app1.docx]

# Appendix 1: Detailed Presentation of Each Case

**Breast Cancer Action (BCA)** is a grassroots organization for women with breast cancer and their supporters; it was founded in 1990 (Table A1). BCA was born from the initiative of Elenore Pred, a victim of breast cancer, who felt that government agencies and organizations provided inadequate and superficial information rather than scientific evidence about breast cancer. BCA works with other organizations to bring about important policy changes on the local, state, and federal levels; it is now a national organization at the forefront of the breast cancer activist movement. They have a staff of eight full and part-time employees.

| Table A1. Breast Cancer Action | |
| --- | --- |
| **ICT Tools:** | **Evidence** |
| - Website - e-newletter - e-mail alert | <http://bcaction.org/> |
| **SM Tools:** | **Evidence** |
| - Facebook - Twitter - YouTube - Blog - LinkedIn | <http://www.facebook.com/BCAction>  <http://twitter.com/#!/BCAction>  <http://www.youtube.com/watch?v=2lT6sV_z5eg> [http://www.thinkbeforeyoupink](http://www.thinkbeforeyoupink.org/) |
| **Main Rationales of SM Use:** | **Evidence** |
| - Provide a voice for breast cancer victims - Awareness and advocacy tool - Promoting campaigns - Build connections with other organizations - Education | *We’ve been historically a kind of an angry organization. The organization was formed from women who were frustrated and angry about the lack of attention that breast cancer was receiving amongst all the cancers and particularly, feeling it was a gendered disease (…) patients should be quieter and more passive and more complacent about women’s responses to it and their diagnosis and it shouldn’t be talked about, and so our initial kind of Facebook and tweets were kind of in that vein, and they have now become viewed with that sense of anger but that anger is now more urgent – more an urgency* |

**The Breast Cancer Society of Canada (BCS)** is a registered, national, non-profit, charitable organization dedicated to funding Canadian breast cancer research into the detection, prevention, treatment and to ultimately finding a cure for the disease that women fear most (Table A2). BCS was founded by Lawrence and Kay Greenway in 1991 after their daughter passed away from breast cancer. They started as a grassroots charity and have evolved into a national organization whose goal is to raise funds for cancer research dedicated to discovering “the causes of breast cancer, better methods to prevent and detect it, treatments that are more effective and improving the quality of life for survivors” ([www.bcsc.ca](http://www.bcsc.ca)).

| Table A2. Breast Cancer Society | |  |
| --- | --- | --- |
| **ICT Tools:** | **Evidence** | |
| - Website-blog - e-newletters - e-mail (email campaigns) - Online coupons | [www.bcsc.ca](http://www.bcsc.ca) | |
| **SM Tools:** | **Evidence** | |
| - Facebook - Twitter - LinkedIn - YouTube - Jumo | <http://www.facebook.com/breastcancersocietyofcanada>  <http://twitter.com/#!/bcsctweet>  <http://www.youtube.com/watch?v=2GPOVCzNb_c> | |
| **Main Rationales of SM Use:** | **Evidence** | |
| - Create awareness about the organization itself at a low cost - Post/re-post online information for readers who access information in different ways - Fundraising - Inform about how the money raised is spent - Imitation (natural evolution of an organization in the modern world) - Be responsive, sensitive - Create a social community that is connected more to their cause and their brand - Reach a target population (young) - Provide information about alternative – complementary treatment - Reach people who are in remote regions - Inform people about specific events | *I think it opens up many doors, myself. It keeps us very modern, it allows us to be very nimble, too, in communicating to people. It allows us to – we quite often will say, “Hey, did you hear about this study today?” And we’ll post it so people get information right away. So, I think it’s a fabulous tool and as far as a cost effective, it’s a very cost effective way to communicate with people too.* | |

**The Canadian Breast Cancer Foundation (BCF)** was founded in 1986 and is a national volunteer-based organization dedicated to supporting a variety of activities around funding cancer research and providing education and awareness program, with the ultimate goal of “creating a future without breast cancer.” (<http://www.cbcf.org>) (Table A3).

| Table A3. Breast Cancer Foundation | |  |
| --- | --- | --- |
| **ICT Tools:** | **Evidence** | |
| - Website - e-newsletters | <http://www.cbcf.org> | |
| **SM Tools:** | **Evidence** | |
| - Facebook - YouTube - Twitter - LinkedIn - Flickr - Blog | <http://www.facebook.com/CanadianBreastCancerFoundation>  <http://twitter.com/#!/cbcf_bcyukon>  <http://www.youtube.com/watch?v=-RWh2Ag4u08>  <http://findinghope.cbcf.org/> | |
| **Main Rationales of SM Use:** | **Evidence** | |
| - Create awareness, bring attention to events - To get involved in the “space” where people were having relevant conversations - Provide a forum for individuals to share stories/contribute to a community - Provide information about events; communicate with individuals who have participated in events - Engage with corporate partners - Complement traditional media efforts in creating brand awareness, etc. - Reach a large number of people at once - Flexible; easy to modify programs/plans | *So, within 2008 we addressed SM and said, ‘This is something that we think is an important area to be involved in, and from there we built out a stronger program.’ So, we started including Facebook, and YouTube, and Blog information in all of our communications, mostly to the Run for the Cure which is our national title event.* | |

**Us Too International (UsT)** is a non-profit prostate cancer education and support network founded in 1990 (Table A4). Their mission is “to help men and their families make informed decisions about prostate cancer detection and treatment through support, education and advocacy” (www.ustoo.org).

| Table A4. Us Too International | |  |
| --- | --- | --- |
| **ICT Tools:** | **Evidence** | |
| - Website - e-mail (e.g. e-blast, e-mail campaign) | [www.ustoo.org/About_UsTOO.asp](http://www.ustoo.org/About_UsTOO.asp) | |
| **SM Tools:** | **Evidence** | |
| - Facebook - Twitter - LinkedIn - YouTube - Wikis - Online discussion communities - Groupon - Blog | <http://www.facebook.com/UsTOOInternational>  <http://twitter.com/#!/USTOOHQ>  <http://www.youtube.com/user/UsTOOInternational>  <https://www.inspire.com/groups/us-too-prostate-cancer/discussion/personal-journey-blog-join-me-in-the-discussion/?follow> | |
| **Main Rationales of SM Use:** | **Evidence** | |
| - Create awareness about the organization at low cost - Post/re-post online information for readers who access information in different ways - Inform people about specific events (repertory of events) - Fundraising - Imitation (natural evolution of an organization in the modern world) - Constitute an advocacy group - Organize online-discussion communities and support group for people who live in remote regions (territory coverage) - Address specific concerns for both patients and families depending on area of interest, stage of disease, or preferred treatment. | *… we’ve never really had a budget for marketing or ads or things like that, so as SM came up, you know, we’ve tried to take advantage of it.* | |

**The Prostate Cancer Foundation (PCF)** is the world’s largest philanthropic source of support for prostate cancer research to discover better treatments and a cure for prostate cancer (Table A5). At the time PCF was founded in 1993, despite its common occurrence, prostate cancer was still the “hidden” cancer and it received little attention from researchers. To give men and their families hope, PCF set out to harness resources—both financial and human—to accelerate the development of new breakthroughs and find a cure as quickly as possible. Their mission is ambitious but very clear: to eliminate prostate cancer as a life-threatening illness for men and their families.

| Table A5. Prostate Cancer Foundation | |  |
| --- | --- | --- |
| **ICT Tools:** | **Evidence** | |
| - Website - e-newsletter - e-mail | <http://www.pcf.org/site/c.leJRIROrEpH/b.5699537/k.BEF4/Home.htm> | |
| **SM Tools:** | **Evidence** | |
| - Facebook - Twitter - LinkedIn - YouTube - Blog | <http://www.facebook.com/PCF.org>  <http://twitter.com/#!/pcfnews>  <http://www.youtube.com/watch?v=hZpvVw55vtE>  <http://mynewyorkminute.org/> | |
| **Main Rationales of SM Use:** | **Evidence** | |
| - Post/re-post online information for readers who access information in different ways - Provide online support & connections to others at a low Cost - Engage individuals through more personal interaction - Platform for having a voice /talking about issues - Reach a different demographic | *And that’s one thing SM is good – I mean -- it is reaching us into the younger group, which is a target for us.* | |

**Pints for Prostates (PFP)** is a non-profit organization founded by prostate cancer survivor Rick Lyke in 2008 (Table A6). It aims at raising awareness among men about the need for regular health screenings to increase the likelihood of earlier detection and PSA (prostate-specific antigen) testing. PFP cooperates with organizations that works with men on awareness, offers support following treatment, or conducts research aimed at improving care or finding a cure for prostate cancer.

| Table A6. Pints for Prostates | |  |
| --- | --- | --- |
| **ICT Tools:** | **Evidence** | |
| - Website - e-mail | [pintsforprostate.org](http://www.pintsforprostates.org/) | |
| **SM Tools:** | **Evidence** | |
| - Facebook - Twitter - Flickr - Vimeo | <http://www.facebook.com/group.php?gid=105465930295>  <http://twitter.com/#!/pints4prostates> | |
| **Main Rationales of SM Use:** | **Evidence** | |
| - Awareness tool - Promote events (Bulletin Board for events) - Build connections with other organizations - Education - Build a community of volunteers | *So, we were – we were just sort of like a little bit of a movement and, just knowing where the world is going these days as far as media goes and how – how people consume media. That just meant that SM was – was almost a given for us. It wasn’t like we had to be convinced about it or had to discover it. It was all around us when we launched.* | |
