# Supplementary material for: Social Media Strategies for Health Promotion by Nonprofit Organizations: Multiple Case Study Design
Source: J Med Internet Res. 2020 Apr 6;22(4):e15586. doi: 10.2196/15586 (PMC7171585; doi:10.2196/15586)
Supplement: Multimedia Appendix 2 [file jmir_v22i4e15586_app2.docx]

# Appendix 2: Data collection tools

**DATA DOSSIER**

**Structured data collection tool for websites and SM tools for each organization.**

Data collection tool for each website

*Record information using copy and paste from the original website or SM tools*

Organization name

Mission ; Specialties; Priorities

Programs and Services

Tools available

Partners and sponsors

Photos

Events

Notes

Causes

Discussion

Main topics

Noteworthy

Data collection tool for each SM E.g. Facebook pages (Excel)

- General information: Organization (owner of the Facebook page), post number, comment number, date, username, user gender.
- Responses to each post or comment: Number of like, number of shares, category of users (individuals or organizations), identity of the user (patient, family-friend, volunteers, organization owner, unknown individual, research and healthcare organizations, other organizations-specify, healthcare professional-specify, unknown organization)
- Issues addressed: Screening-preventing, treatment, support and general coping strategies, prognosis and end of life, research, policies, specific event, other-specify)
- Purpose of Facebook posting: Educating-informating, sharing stories-personal anecdotes-testimonials, supporting, simply agreeing, advocating, raising funds, requesting information, thanking, other-specify
- Stance: Initiating, responding by providing additional information, simply supplementing, debating, other-specify, sharing photos.

**Interview guide**

**Introduction:**

[Don’t need to say all of this if they’ve read the consent form already].

First, thank you for agreeing to meet with me in spite of your busy schedule. We are conducting research about the role of SM in health promotion, particularly in the context of breast and prostate cancer awareness.

This is a request for, at most one (1) hour of your time for a free ranging discussion of these issues. You are free to participate or not in this interview and you can stop at anytime.

The conversation will be recorded to ensure the accuracy of the data collection. The analysis of our conversation will be kept anonymous and strictly confidential; unless you explicitly allow us to do so, there will be no way to identify respondents or to relate them to their commentaries.

Please take the time to carefully review these important points included in the *consent form*, which has been faxed to you earlier. Do not hesitate to ask any questions or raise any concerns you may have with any of the items contained therein.

First, we will ask you a general question about the use and the evolution of the use of SM in your organization for health promotion. Then we will discuss more specific aspects relating to the strategies and impacts of using SM in your organization.

**Part 1: History**

1. [No more than 20 minutes] Can you tell me how your organization became interested in the use of SM? By SM, I mean tools such as Facebook, Twitter, Youtube and the like.

[Note: if necessary, direct the conversation towards the use of SM for awareness/promotion – not for fund raising, unless the two are inter-related]

Prompts:

- When did the idea to use SM in your organization first come up?
- What kind of SM do you use?
- For what purposes/how do you use it?
- Why do you use it? (rationale)
  - Is the way you use SM complementary or supplementary to your traditional strategy? (Winer 2009)
- Who initiated the use of SM in the organization?
  - Was anybody particularly involved in the promotion of the use of SM?
  - What rationale did s/he provide?
- Did you encounter any particular challenges in implementing your SM strategies?
  - What types of challenges?
  - Was the need for additional resources (human, capital, etc.) an issue?

**Part 2. Strategies**

Now, I would like to understand better how your organization achieve its goals of health promotion. If we think about it, there are two different types of people, right?: Those who seek information from your organization vs. those you would like to reach.

1. Do you have different strategies for reaching these different groups of people?
2. [OPEN ENDED] Thinking about the people who pro-actively seek information from your organization. What do you do to meet their needs?

[IF THEY DON’T MENTION] I’ll name some things – please tell me if you’re doing any of these things.

- Newsletters – Offline
- Newsletters – Online
- Partner with experts who can provide credible information
- Surveys/Polls
- Posting information on your website

1. [OPEN ENDED] Thinking about the people who you would like to reach, who are not pro-actively seeking you out. Can you tell me what your organization does to reach this group of people?

[IF THEY DON’T MENTION] I’ll name some things – please tell me if you’re doing any of these things.

- Traditional advertising (TV, Radio, Newspaper, etc.)
- Online advertisements
- Partnerships (with bloggers, experts, other similar organizations)
- Sponsorships (sponsor events)
- Mobile advertisements
- E-mail campaigns
- Public speaking
- Create word-of-mouth
- Search engine optimization
- Invite customers to create their own advertisements for the company (Winer 2009)

Now I would like to talk more specifically about different types of SM that you may or may not be using. For each of the tools, I will ask you a few specific questions.

|  | | **Do you use this?** | **When did you start using _____?** | **Why do you use ______ in particular?** | **How do you use ______ in particular?** | **Pull strategies: How do you use ____ to meet the needs of people who are seeking information from your organization?** | **Push strategies:**  **How do you use ____ to reach those who do not pro-actively seek information from your organization?** |
| --- | --- | --- | --- | --- | --- | --- | --- |
| **Facebook** | |  |  |  |  |  |  |
| **Twitter** | |  |  |  |  |  |  |
| **YouTube** | |  |  |  |  |  |  |
| **Website** | |  |  |  |  |  |  |
|  | **User Feedback (Review System)** |  |  |  |  |  |  |
|  | **Discussion Forum** |  |  |  |  |  |  |
| **Blogging** | |  |  |  |  |  |  |
| **Other tools** | |  |  |  |  |  |  |

**Part 3. Impact of these strategies**

In the next part, I will ask you questions about the impacts of the SM strategies you have described to me.

1. Do you think your SM strategies are working to meet your organization’s health promotion goals?

- Are your SM strategies helping to meet the needs of those who pro-actively seek information from your organization?
- Are your SM strategies help you reach those you would like to reach, who are not pro-actively seeking you out?
  - Have you increased your reach since you have started using SM?
  - Have you been able to reach a different market/population?
  - Are you able to reach people earlier in the course of the disease?

1. Are these strategies useful to meet any other of your organization’s goals?
2. How do you assess success of your SM use?

- Do you feel that implementing the SM strategy was worth the resources invested?

1. Plans for future development of SM strategies?
   - Evolution in strategies?
2. Reflecting on the implementation of your organization’s SM strategies, if you were to do it again, is there anything you would do differently?
   - Were there any challenges that you felt you faced in implementing the SM strategy (losing control, etc.)?

**Part 4: Wrap-up**

This concludes the questions that we have planned to ask you today.

Is there anything else that you feel is important to mention relative to the use of SM?

Is there anything else we did not raise that you think is relevant to mention in the context of this study?

1. Is there any other information, such as an annual report or any other documentation, that you would like to provide that you think we may need?
   1. Annual report
   2. Activity report – documentation
   3. Number of people who adhere
2. Finally, is there someone in your organization who was involved in the technical side of the SM implementation who I could possibly contact?

**Socio demographic questions**

This socio demographic information is being collected solely for the purpose of data analysis in the context of this study. This data will be kept anonymous and strictly confidential. Unless you explicitly allow us to do so, there will be no way to identify respondents.

1. Profession / background: Since? _ _ _ _(yyyy)
2. Place of employment / industry? : Since? _ _ _ _(yyyy)
3. Position(s) occupied(s)? : _____

We thank you for your participation. Please note that the time you spent with us is much appreciated and that your comments are of great importance for the success of our study.
