# Supplementary material for: Social Media Strategies for Health Promotion by Nonprofit Organizations: Multiple Case Study Design
Source: J Med Internet Res. 2020 Apr 6;22(4):e15586. doi: 10.2196/15586 (PMC7171585; doi:10.2196/15586)
Supplement: Multimedia Appendix 3 [file jmir_v22i4e15586_app3.docx]

# Appendix 3: Coding scheme

1. **Initial coding: codes from organizing vision**

*General information on organization:*

- name, type of organization, country, disease type; year founded; number of employees;
- Mission ; Specialties; Priorities
- ICT used: General purpose; Goals; SM Tools Used (purpose and tools main characteristics)

*Comprehension*

- First use of SM
- SM in general: General purpose-rationale; Goals (Pull vs. push information) e.g. provide general info to people who are searching info; Provide individualized answers
- Key drivers that led the organizations we studied to adopt a SM strategy: e.g. Provide a voice for breast cancer victims; Awareness and advocacy tool; Promoting campaigns; Build connections with other organizations; Education
- Issues addressed: E.g. Screening-preventing, treatment, support and general coping strategies, prognosis and end of life, research, policies, specific eventSM Tools Characteristics - opportunities. e.g. quiz that can be the base of a word-of-mouth; Individualize the information provided to what people are interested; conversation with people – Responsiveness - Rapidly provide information; Allow to reach people who are not looking for information (word of mouth),

*Adoption*

- Strategies put in place. E.g. By knowing the behavior of people, it is possible to target the message (e.g. donation);

*Implementation*

- Complementary (information, events, image, video)
- Challenges: e.g. Requested expertise, learning by doing

*Assimilation*

- Main impacts associated with the use of SM in that organization.
  - Expected Impacts: Increase awareness about disease; Enables fundraising; Support and educate users.
  - Unexpected impacts: Increase awareness and visibility for the organization; Reach new audiences; Target messages more effectively; Engage users; Allow users to interact and provide feedback; Co-create knowledge; Create networks of users

1. **Second round of coding (developing categories and themes)**

- *Rationales for adopting and using SM*: Creating awareness; Educating; Supporting; Advocating; Raising funds
- *Opportunities that are enabled through SM:* Interactivity; Flexibility; Ease-of-use; Low cost; Trendiness; Virability
- *Strategies that organizations enact to make the most of SM:* Replicate; Transform; Innovate.
- *Complementarities* between ICT and SM tools.
- *Challenges that the use of SM creates:* Lack of Control; Technology-Related Issues; Diversity of Audience; Availability of Resources; Difficulty in measuring impacts
- *How strategies were enabled****:*** Reactive/Proactive/Mindless/Mindful**;**

1. **Third round of coding. Link between categories. Theoretical framework**

- We developed the dynamics of the process and the links between the previous categories.
