# Supplementary material for: Social Media Strategies for Health Promotion by Nonprofit Organizations: Multiple Case Study Design
Source: J Med Internet Res. 2020 Apr 6;22(4):e15586. doi: 10.2196/15586 (PMC7171585; doi:10.2196/15586)
Supplement: Multimedia Appendix 4 [file jmir_v22i4e15586_app4.docx]

# Appendix 4: Quotes from Key Informants

Abbreviations

BCA: Breast Cancer Action

BSC: Breast Cancer Society

BCF: Breast Cancer Foundation

UsT: Us too International

PCF: Prostate Cancer Foundation

PFP: Pints for Prostate

| **Section 3.1: Comprehension** | |
| --- | --- |
| Creating awareness | |
| 1 | *Facebook, it seemed as a way to reach a broader segment of the population. There is the opportunity through Facebook to reach a broader demographic. BCA* |
| 2 | *Where it’s a little different is that we have such an immediate and active community in terms of journalists that are there or organizations that want to get involved. It seems like there’s a lot more of discussion that’s kind of back and forth with Twitter, which has been beneficial for us whenever we are talking about events and there are interested parties that connect us to other opportunities. BCF* |
| 3 | *We’re still feeling our way and trying to figure out, you know, -- not trying to alienate our general audience who – the people who are usually looking for prostate cancer information, but making sure – you know -- while we try and reach, you know, the sons and daughters of men impacted by prostate cancer. Those are things we’re looking at. How also can we talk to these -- that generation? And that’s one thing social media is good – I mean -- it is reaching us into the younger group, which is a target for us. PCF* |
| Educating | |
| 4 | *So, one of our kind of missions is to provide women with the information that they need to make informed decisions for themselves. BCA* |
| 5 | *Through Facebook she’s addressing questions and even things that people might not think to ask, but it’s good to know. Like why are there so many breast cancer organizations? It’s a great way to address concerns or to throw the facts out there or get people thinking in a very easy way. BCS* |
| Providing a forum to interact and support | |
| 6 | *We have had people ask for certain things because we let everyone comment. So, that for us is wonderful, because that’s what we want. We want to know what they need from us and if we can’t help them how we can direct them and we have a lot of really valuable resources available to anyone looking for information. BCS* |
| 7 | *We also have a listing of all of our support groups everywhere, so that’s great if there’s one in your city, but if there’s not one in your city we have, you know, virtual supports groups, if you will, through our online discussion groups. UST* |
| 8 | *A lot of times people think that as a disease organization, we’re similar in structure to some of the larger organizations, so they think we have more resources to provide kind of online support or connections to others, you know, with the same disease and we just don’t have it. That’s where social media has been helpful because we connect those people through those tools. PCF* |
| Advocating | |
| 9 | *We’ve been historically a kind of an angry organization. […] the organization was formed from women who were frustrated and angry about the lack of attention that breast cancer was receiving amongst all the cancers and particularly, feeling it was a gendered disease. And so, […] our initial kind of Facebook and tweets were kind of in that vein, and viewed with that sense of anger. BCA* |
| 10 | *We advocate – we want guys – our advocacy is for guys to get tested and, you know, the challenges that have kind of come to us from SM is – is that we’re out there in a world where when you are talking about subjects like prostate cancer. There’s a ton of misinformation about it. PFP* |
| Raising Funds | |
| 11 | *Last year we added a little badge to our Run for the Cure website off of our main website to Facebook, and by adding that connection from Facebook to the participant or the person getting involved with the run, we know we garner to about 70%. 70% of them are new donors and new participants. So, we know that we’re reaching a new audience that way. BCF* |
| 12 | *And then also it is a mechanism to let people when we have national events so that they can participate. If we have a webinar or if we have a new resource or if we have a fundraiser event, like a walk or something like that. UST* |
| 13 | *The Great American Beer Festival has 50,000 people who come through their doors during four sessions. (…) we’ve done this event now for two years. (…) Last year, it was 500 people and we charged $80 a ticket. We sold all of our tickets online. We promoted it almost exclusively last year with social media. PFP* |
| Ease-of-Use | |
| 14 | *Facebook has, well the easiest application really and the percentage of people who use it is astronomical. So, that’s probably our best tool to reach as many people as possible (...). I think Twitter is probably the next user-friendly sort of well-known SM tool available. It ties in nicely with Facebook actually. BCS* |
| Low Cost | |
| 15 | *We’ve never really had a budget for marketing or ads or things like that, so as SM came up, you know, we’ve tried to take advantage of it. UsT* |
| 16 | *I mean – it takes some effort but – you know, they are low cost. We are able to repurpose content and we like to also use them to start discussions that we couldn’t do in person or, you know, within chapters. PCF* |
| Interactivity | |
| 17 | *Everyone saw it as a new way of communicating. We, for many years, have been gathering email addresses and we would send out email blasts sometimes. I guess you can have more of a conversation with many people when you’re using Facebook and Twitter as opposed to straight emails back and forth. BCS* |
| 18 | *We found that people do like to see the news but they are much more engaged, of course, if you make it personal to them—either through a personal story or ask a question that they can respond to. So it’s the usual progression from kind of just writing these stories and posting them there to try to come up with more creative ways to get people to respond or comment or interact with us. PCF* |
| 19 | *If we’ve got thirty people around North America who are interested in doing events, that’s the easiest way for them to be reminded of us and to see what’s going on elsewhere in the country. We’ve got our webpage and they can go there, but it actually means they have to click on it and go look. With a Facebook page or with a Twitter feed, we’re doing outbound to them. PFP* |
| 20 | *…we quite often will say, “Hey, did you hear about this study today?” And we’ll post it so people get information right away. BCS* |
| Flexibility | |
| 21 | *We are trying to apply old media usage standards to this new technology and we should just throw it all out the back door because we need to rethink – it’s all so new and dynamic and changing that maybe it’s as opposed to committing to conventions that already exist – perhaps with Facebook we actually get to define some of the conventions like when we put the billboard up. But it’s a lot more freeing than perhaps we are giving it credit for. BCA* |
| 22 | *Our YouTube is a repository for any promotional videos or public service announcements that we put out. So, we put them all in the same place then whenever we’re linking content through our SM networks, or through the website, we link it all through YouTube, so it’s sort of an easy platform to coast all of those videos. BCF* |
| Status | |
| 23 | *I think it opens up many doors, myself. It keeps us very modern, it keeps us -- it allows us to be very nimble, too, in communicating to people. BCS* |
| 24 | *Well, number one – it’s the hottest thing out there. (…) We might as well use this that’s out there – that everybody else is using. UsT* |
| Virability | |
| 25 | *It’s a quicker, more efficient way and, you know, obviously we are reaching people on their mobile devices and there’s definitely a kind of – viral, almost like rabid use of mobiles.- about Twitter BCA* |
| 26 | *That stuff just virally spread. We had a video that a company shot during the first year and we sent that out to all of the people who attended the first year and then we’ve – we’ve also – they have it up on a site. We’ve got it on our page. Their site alone – we’ll it’s up on – I think it’s a service called T-Bow. Thirteen or fourteen hundred people went in and looked at that – and that doesn’t count the people who saw the link that we shared with [T-Bow]them or watched it on our site. PFP* |
| **Section 3.2: Adoption** | |
|  | Use of SM tools in addition to ICT tools and more traditional communication tools |
| 27 | *It helps them that we are in there in the prostate cancer space and so we may promote the three guides we have or our newsletter on Facebook or point them to our site so that they know and that’s just one way – if they’re just searching around, it’s almost like another search engine and they can find out who’s in the prostate cancer space and find out what kind of resources there are. PCF* |
| 28 | *We don’t generally use Facebook for telling the whole story; we’d rather use it for giving a little bit of information then offering them next steps to get back to the website, or to connect with us directly to get the full story. Just because Facebook is a limited platform, just like Twitter, you don’t have the room nor is it the place to really tell. So, we always direct that back to the website, it’s sort of a stamp in the road (…) Because it’s so short, it’s more of a conversation builder and we’re trying to build out our awareness level up there, but in terms of sharing really complex news, that’s now what we’re using it for. We’re using it for more of a community building aspect. BCF* |
| 29 | *Another fundraising thing that’s connected to this is online coupons. So, here you go connecting with people you didn’t really connect with before and allowing us to make a connection where they are basically shopping for a coupon, hey by the way here’s our logo and here’s our website, and connecting to them that way. So, that’s unique because in the world of fundraising you’re always looking for a new way to raise money that is not necessarily traditional. BCS* |
| 30 | *It vastly increases the number of signatures we can get because our alternative is to hit the streets, send e-mails out and have people e-mail responses. So what we can now do is partner it with an e-mail campaign and a Facebook campaign and we’ve got a double-whammy component. BCA* |
| 31 | *I’ve tried to create kind of like a cheater’s simple blog on our website with our news and events column…Not all of these posts make it onto our Facebook page (…) Well, we do e-blasts. People who are – are friends, donors, volunteers, people who have sought information from us in the past and, you know, new subscribers – and we’ll do e-blasts or postings on our various discussion group lists. UsT* |
| 32 | *We also have our Run for the Cure, which is how we’ve basically built the page to what it becomes today. So, our biggest [Facebook] fan base is through the run because we communicate with them... So, we believe that is how our numbers have grown so significantly. BCF* |
| Replicate | |
| 33 | *We have our basic message about the importance of men getting tested starting at forty and, you know, under our old group we would – we would look for articles and things that – that made that point and we would paste them to our, you know, to our Facebook group so that our members could see them and then, you know, we would share – ask them to share them with their friends (…) I put up there the other day some video from Good Morning America from Erin Andrews and her dad who, you know, is a prostate cancer survivor. So, we try to use it as a – as a reinforcement tool so that people who come and visit the site, you know, see that this is , you know, a disease they need to pay attention to. PFP* |
| 34 | *So, primarily – well, on our website, of course, we produce our news ourselves – so at the outset we initially were using Facebook and Twitter to kind of post – repost that information and, you know, get the news out there on another forum for readers who, you know, prefer to read it on another venue. And also Dan -- Dan has started his own blog and we like to repost content from that blog on both of those. PCF* |
| Transform | |
| 35 | *We do use to try to promote our events and one good thing about Twitter is that, you know, if you effectively use hashtags and add symbols, you know, you can tend to reach people outside of your network. You know, so we use it as an outreach tool to try to – to try to make people aware of events who might not be beer geeks, who might not be a member of our Facebook page, or check our website regularly. PFP* |
| 36 | *The last Valentine’s we did it also on Facebook. So people could – it’s a way of, again, a way of breast cancer awareness, impending it on to your friends, and thinking about the people that have been through this disease and how you’re thinking about them at this time. So, we kind of used it that way. We just started adding things like questions and quizzes to kind of get people engages and try to get people to forward things on. BCS* |
| Innovate | |
| 37 | *…a woman whose husband was diagnosed with prostate cancer last year in Wisconsin – they owned a car dealership and they were going to do an ad and she said, “We want to do an ad for prostate cancer awareness,” and so she videoed it and sent it to us and we posted it on Facebook. So, it was like a – like a commercial for awareness and prostate cancer, you know, go get tested if you have it in your family type of messaging…and we try and post these things to give people ideas that you can do this too. UST* |
| 38 | *I think that the reason – well from my perspective and from what I know about the way that we interact with our communities and SM, what’s been so successful is that we share a range of information and often share compelling and engaging stories. Storytelling is a major part and major component of all of our SM and planning and objectives that we get the most traction and most interest and most conversation whenever we share information from people that have an engaging story or an inspiring story related to breast cancer, or related to how they’ve worked with us in terms of researcher or volunteers. So, for us that’s the strongest way we’ve engaged with our public. BCF* |
| **Section 3.3: Implementation** | |
| Lack of Control | |
| 39 | *Overseeing the content of the website – I was a little concerned that there would be some worry about the loss of control or the content once we had this kind of more open platform that would happen. And so, it was very much a process of making sure it was followed – we were posting first. It was more formal. Then slowly people became more comfortable with it as being different and within organizations they are more comfortable with different topics being talked about and just understanding that people are going to comment and other people understand that sometimes those comments don’t work like the organization. PCF* |
| 40 | *I see postings where people have the same old myths that you would encounter in the analog world are out there in the digital world. The only problem is the digital folks can reach a hundred people where the analog people are only talking to one person and giving them bad information. PFP* |
| 41 | *I think the biggest issue we had to get through when we were establishing how much we were going to be engaged in SM was addressing issues management and reputation management. If conversations are happening without complete, complicit, control how would we manage attacks, negative information, or incorrect information that was being circulated about us. In the end, we came to a place where we had to decide that we were either going to get involved, allow this to happen, then manage as we saw fit, or not get involved and miss out on a major opportunity. BCF* |
| 42 | *We had an event with bands and stuff at our Hard Rock Café restaurant here...they created a Facebook page which we liked or friended or whatever, and then we – they also have a Twitter page. So they tweeted a lot of stuff and so did the run/walk event (…) Frankly, some of the tweets, I thought, were getting kind of annoying because they were just sending out so many things in one day it was just – it just seemed kind of silly and it wasn’t necessarily new or really interesting information.” UsT* |
| Technology-Related Issues | |
| 43 | *The one thing that Facebook has done recently that makes it even more difficult is that they’ve changed – and this has me scratching my head as to, you know, what their motivation is (…). So, on Facebook – if you and I were friends, six months ago every time you posted I would see what you posted. Okay? The way they’ve changed things, if you and I haven’t communicated back and forth directly to one another – like, if I haven’t liked one of your posts or if you haven’t commented on one of mine, now I’m not going to see your posts anymore (…) This whole thing that they’ve done means that fewer and fewer people have the chance to become exposed to our message. It’s a lot less viral. They’ve inoculated people so that you only, you know, it’s only like your little village that you’re constantly in contact with that you see unless you open it back up again. PFP* |
| 44 | *Twitter is only great for getting catchy conversation and generating interest and driving conversations back to the blog or our Facebook, or our website. Because it’s so short, it’s more of a conversation builder and we’re trying to build out our awareness level up there., but in terms of sharing really complex news, that’s now what we’re using it for. BCF* |
| Diversity of Audience | |
| 45 | *We see more fifty and sixty year olds tweeting, perhaps, and Facebooking; but, we need to reach the younger demographic in terms of they’re the ones who are assuming caretaker roles and they are the ones who are being more proactive and understand the need to (a) be proactive about a prostate health plan and (2) are open to talking about as a group. Older folks just don’t want to talk about it – they want to push it under the rug. PCF* |
| 46 | *So, the actual messaging and how we deliver that information has to be rethought., (…) we have to really re-think how we do that (…). I’m going to guess that the non-English speaking, you know, immigrant labor force that’s perhaps undocumented – I don’t know how many of those people are on Facebook. BCA* |

| Availability of Resources | |
| --- | --- |
| 47 | *It would just be around managing the time for us, which worked really well that we had a dedicated staff person at our central office who has been –their focus is managing our SM space and originally it was volunteer role and it became such a high volume position, we actually have a staff person engaged in that job… BCF* |
| 48 | *We probably have spent, I would say a disproportionate amount of time overall on SM but, it’s – the other thing that, you know, about it is that if you’re not constantly feeding it, you know, it’s sort of like this ticker tape (…) if you don’t keep feeding this ticker tape thing, somebody could get there ten minutes later and they don’t see your message. So we’ve – we’ve spent more time there probably than most other things. But we get a return for it, so we keep doing it. PFP* |
| 49 | *So what we would do is we would try to – say, okay, we had a bird. and it would actually – if you pressed a button it would actually – it would start tweeting. And whoever had that bird on their desk was responsible for tweeting posts that day. Which completely undermined the kind of spontaneous advocacy and potential of Twitter which was, at a particular given moment throughout the day when something occurred to somebody that was relevant to the debates and issues around breast cancer that are specific to Breast Cancer Action – they would spontaneously take the initiative to tweet about it. BCA* |
| 50 | *I think that’s probably finding the right person that could do that (…) I’ve got a LinkedIn page and I’ve signed up to different groups. I must confess though, I probably need to spend more time with that. That’s kind of where I would go with partnerships. BCS* |
| Difficulty in Measuring Impacts | |
| 51 | *I know with Facebook or SM it’s challenging because we don’t have data about the people that are engaging with us. It’s often we don’t even know, we can sometimes know where they are from, etc., but we don’t know what we know for something like a donor or participant, .we know about them and where they are from and their information and how they are engaged. It’s a little bit more anonymous in that way, so it’s harder to say (…). We internally have the ability to understand our numbers, but we’re wondering how are we comparing to other organizations of the similar nature. BCF* |
| 52 | *It’s a little hard to measure and it takes a lot of time to measure. PCF* |
| **Section 3.4: Assimilation** | |
|  | Mindless/Mindful |
| 53 | *And he (one of their volunteers) created a Facebook page, so he was more of an early adopter. And then once we saw all the activity on his page we thought, “Uh-oh, we’d better have a Facebook page too.” So, that’s how we set up our Facebook page and then after that we also created a Twitter account; but, frankly, we don’t really use it very much. And then another volunteer just out of the blue came up and said, “Oh, well, I’d be happy to create a My Space page for Us TOO,” which she did. UST* |
| 54 | *It was -- it was like the web blast of, oh – fifteen years ago – Everyone knew they had to do it, but no one was quite sure why. It was the directive, “Okay, we need to be here”, and it was a little bit of, “Okay – we will get there and we will define as we get into the waters and we find our strokes and stay afloat”. PCF* |
| 55 | *I think once again this falls a bit in the category of this is all so new. So, I think we’ll learn over time; but I don’t think it’s strictly understood or defined yet (…) We’re just constantly assessing where we are getting traction, where we are getting engagement, and that’s where we’ll decide to turn things on. PCF* |
| 56 | *And that came, I think, as you know – you know, because we’re in the Bay area – there’s various kind of partners of people who work for the organization or in the Tech industry and are familiar with how Facebook and Twitter are used professionally. and the idea that people kind of have is it a – in the professional world that Facebook and Twitter is used in a more mature way to follow professionals who are engaged in the conversations that you are engaged in. And so we are moving a little more, I think, to kind of that model of both a sense of urgency and action but also keeping people up to date on our take on conversations and policies and issues to do with breast cancer that are happening, you know, on and off throughout a month or a week. BCA* |
| 57 | *That was sort of how we started and then from there we built, watched trends, and monitored activity to see where we should be moving to next, and then just built it out from there (…).I think we’ve had great success so far. We keep building (…)We just hope to keep building on that and keep expanding to spread awareness about the organization. It’s an ongoing thing, it’s flexible, but that’s what is so great about SM is that we’re able – it’s such a flexible space that we can modify programs or plans with SM as we move along. BCF* |
|  | Reactive/proactive |
| 58 | *Around about the time we started to use SM was when we discovered that people wanted to have an easy way to be updated., you know, and we were getting asked, “Hey, do you guys have a Twitter feed, do you guys have a Facebook page” (…) PCF* |
| 59 | *For the most part, in the beginning, we wanted to get into the space because we knew that’s where people were having conversations. Whether it’s about breast cancer, or getting involved in the Run for the Cure, or just getting out in their community, we knew this was somewhere we had to have a place (…) BCF* |
| **Section 3.5: Connecting the dots** | |
|  | Cluster 1 |
| 60 | *That just meant that social media was – was almost a given for us. It wasn’t like we had to be convinced about it or had to discover it. It was – it was all around us when we launched.PCF* |
| 61 | *The benefit of such a strategy mainly resides in allowing the organization to reach out to new audiences but does not entail transforming the message or modifying the existing ICTs strategies. As such, the value derived is mostly through enlarging its audience.* |
| 62 | *You know, at 3,500 people we’re going to have to create a page sooner than later, because once you get to 5,000 as a group you can’t invite everybody to events. So the page, what it does, is allows you to geographically target the invitations PFP* |
|  | Cluster 2 |
| 63 | *We found, you know, with a lot of organizations and even, maybe corporations, we found that people do like to see the news but they are much more engaged, of course, if you make it personal to them—either through a personal story or ask a question that they can respond to. So it’s, you know, the usual progression from kind of just writing these stories and posting them there to try to come up with more creative ways to get people to respond or comment or interact with us. PCF* |
| 64 | *Based on the fact that we had a lot of people – staff members who were actually using it and obviously a lot of other organizations were using it, and for us it became a means to stay in closer contact with our members and a more effective tool when we needed a quick turn-around in terms of taking action (…) We didn’t want it to be -- to be kind of, you know just --the words escape me – but, you know, the kind of frivolous, “Hey, I’m in the shower”, “Hey, I’m walking down the street”. (…) So what we would do is we would try to -- say, okay, we had a bird and it would actually – if you pressed a button it would actually – it would start tweeting. And whoever had that bird on their desk was responsible for tweeting posts that day. BCA* |
| 65 | *And I think the potential for us to link the narrative to the visual and to do and, you know, the videos and the YouTube stuff – but, we haven’t gone that route yet in terms of – and I think that’s more to do more with just our resource capacity, that technical capacity in terms of creating and making a video. The video is still the same – a video still has to be played, whereas an icon on Facebook is there for everybody to see regardless of hitting a play button. BCA* |
| 66 | *I think it’s brought in some – new audiences from what we can see. It’s a little hard to measure and it takes a lot of time to measure; but, it definitely helps us – it helped me gauge what – have a quick gauge of what people are interested in, topic wise and we have seen an increase in online giving. PCF* |
|  | Cluster 3 |
| 67 | *That was sort of how we started and then from there we built, watched trends, and monitored activity to see where we should be moving to next, and then just built it out from there.(…) It’s an ongoing thing, it’s flexible, but that’s what is so great about social media is that we’re able – it’s such a flexible space that we can modify programs or plans with social media as we move along. BCF* |
| 68 | *The last Valentine’s we did it also on Facebook so people could – it’s a way of, again, a way of breast cancer awareness, impending it on to your friends, and thinking about the people that have been through this disease and how you’re thinking about them at this time. So, we kind of used it that way. We just started adding things like questions and quizzes to kind of get people engages and try to get people to forward things on. Sometimes, when we’ve had large donation announcements we’ve posted researchers talking on YouTube and put that out there, and also on Facebook, so that donors can have a chance to hear what the researchers are working on and exactly where their donation dollars go. I think those are good strategies too. (…) Now Facebook has added a great feature where you can ask a question and there’s a poll feature. Recently, we did a scavenger hunt type quiz and we find people are engaged with things like that. They have a lot of interest. BCS* |
| 69 | *I think one of the things – well from our perspective we’re really interested to see how one of the growing aspects of mostly our Facebook and social media programming is measuring the impact back to the organization. For us, we’ve been able to measure it through traction and also programs where we had a direct call to action and then we knew that we received pick up based on that. One of the areas we haven’t been able to find much research on is how much donation traction has occurred or has been facilitated through social media engagement. So, that’s one of the areas that this study or similar studies that we’d love to learn more about. BCF* |
| 70 | *I think it opens up many doors, myself. It keeps us very modern, it keeps us -- it allows us to be very nimble, too, in communicating to people. It allows us to – we quite often will say, “Hey, did you hear about this study today?” And we’ll post it so people get information right away. So, I think it’s a fabulous tool and as far as a cost effective, it’s a very cost effective way to communicate with people too. BCS* |
