# Supplementary material for: Social Media Strategies for Health Promotion by Nonprofit Organizations: Multiple Case Study Design
Source: J Med Internet Res. 2020 Apr 6;22(4):e15586. doi: 10.2196/15586 (PMC7171585; doi:10.2196/15586)
Supplement: Multimedia Appendix 5 [file jmir_v22i4e15586_app5.docx]

# Appendix 5: Data Dossiers

Abbreviations

BCA: Breast Cancer Action

BSC: Breast Cancer Society

BCF: Breast Cancer Foundation

UsT: Us too International

PCF: Prostate Cancer Foundation

PFP: Pints for Prostate

| **Tools** | **Indicators** | | | **BCA** | **BCS** | **BCF** | **UsT** | **PCF** | **PFP** |
| --- | --- | --- | --- | --- | --- | --- | --- | --- | --- |
| **Facebook** | | | | | | | | | |
|  | Number of people liking | | | 157 | 36217 | 36970 | 1203 | 13759 | 3530 (open group) |
|  | Number of people talking | | | 3 | 555 | 245 | 3 | 149 | Not reported |
|  | Number of wall posts by organization (1/8/2012-1/22/2012) | | | 0 | 10 | 10 | 0 | 5 | 0 |
|  | If no post, date of last post | | | N/A | - | - | December 28 | - | October 14 |
|  | Mean Number of shares per post | | | No post | 8.1 | 1.4 | No post | 1 | Not reported |
|  | Number of wall posts by others; characteristics of posters (1/8/2012-1/22/2012) | | | 0 | 7  2 “com-mercials”  2 patients  1 daughter  1 sister  1 husband | 10  All “com-mercials” | 4  1 “com-mercial”  1 patient  1 friend  1 relative | 23  14 “com-mercials”  2 patients  3 wives  4 daughters | 0 |
|  | Number of discussion topics | | | 0 | 0 | 0 | 0 | 0 | 0 |
|  | Moderation | | | 0 | Frequent answers to user’s questions | Frequent answers to user’s questions | Some answers to user’s questions | Frequent answers to user’s questions | 0 |
|  | Other | | | - | 2 videos | 6 videos Yard sale | 0 | 6 videos  Shop (sell products) | 0 |
| **Twitter** | | | | | | | | | |
|  | Number of followers | | | 5475 | 1899 | 4443 | 171 | 246 | 1353 |
|  | Number of following | | | 138 | 1016 | 2081 | 206 | 1724 | 1186 |
|  | Number of tweets (own tweets/re-tweets of others). (1/8/2012-1/22/2012) | | | 10/0 | 8/0 | 37/1 | 1/0 | 15/4 | 25/140 |
|  | Do they use hashtags? | | | Yes – 5 unique | No | Yes – 26 unique | Yes – 1 unique | Yes – 9 unique | Yes – 20 unique |
|  | Do they link to other users @? I think so@... | | | Yes – 7 unique | Online to @YouTube | Yes | Yes (not in the time range we are looking at) | Yes – 25 unique | Yes – 9 unique |
|  | Do they link to websites? | | | Yes – 3 | Yes – 7; some YouTube | Yes | Yes – their own website | Yes – 13 | Yes – 6 + their own website |
| **YouTube / Vimeo** | | | | | | | | | |
|  | | | Number of videos: | 9 | 8 | 41 | 21 | 11 | On You-Tube, but don’t have their own channel |
|  | | | Number of video Views: | 13205 | 1177 | 52380 | 2224 | 2942 |  |
|  | | | Number of Subscribers: | 36 | 6 | 54 | 8 | 0 |  |
|  | | | Moderation | No real activity in comments | No comments | Yes | Not a lot of comments | No comments |  |
| **LinkedIn** | | | | | | | | | |
|  | | | Number of followers | 18 | 18 | 637 | Group not company, so info. not available. | 125 | No Linked-In. |
|  | | | Number of employees on Linked In | 10 | 6 | 198 |  | 30 |  |
| **Blogs** | | | | | | | | | |
|  | | | Number of posts (1/8/2012-1/22/2012) | 0 | No blog (it is a website-blog, see website) | 2  1 women with cancer  1 son | 50  Mostly from patients and relatives (support community)  Organized in discussion topics | 4 | No blog |
|  | | | If no post, date of last post | December 29 |  |  |  |  |  |
|  | | | Mean Number of comments | No post | No blog | 2 | 5 | 3.75 | No blog |
| **Flickr** | | | | | | | | | |
|  | | | Total Number of photos uploaded (number of items) | NA | Na | CBCF Ontario - 448 | NA | NA | 182 |
| **Cross-references (SM / ICT)** | | | | | | | | | |
|  | | From Twitter to … | | Website and other research centers/news sites | News articles, videos, and their own website | Website from twitter as well as links to videos, pictures, etc. | Website and the news portion of their website from twitter | Externally hosted documents, news; not really to their own site except in the header | External content (news), events (and other context through the RTs). |
|  | | From website to … | | Facebook and Twitter | Facebook, Twitter and YouTube | Twitter, Facebook, YouTube, blog | Facebook, Twitter, YouTube, LinkedIn | Facebook, Twitter, LinkedIn | Facebook. Twitter feed, but not working |
|  | | From Facebook to … | | YouTube, website, blog | Website, Flikr, YouTube | Website, blog | Website | Website / Twitter | Website |
|  | | From blog to … | | Website |  | Website, Facebook, Twitter, YouTube | Website | Website |  |

Numbers as per January 23^rd^, 2012 or as specified.
